# Observation of Coulomb gap in the quantum spin Hall candidate single-layer 1*T*’-WTe_2_

**DOI:** 10.1038/s41467-018-06635-x

**Published:** 2018-10-04

**Authors:** Ye-Heng Song, Zhen-Yu Jia, Dongqin Zhang, Xin-Yang Zhu, Zhi-Qiang Shi, Huaiqiang Wang, Li Zhu, Qian-Qian Yuan, Haijun Zhang, Ding-Yu Xing, Shao-Chun Li

**Affiliations:** 1 National Laboratory of Solid State Microstructures, School of Physics, Nanjing University, 210093 Nanjing, China; 2 Collaborative Innovation Center of Advanced Microstructures, Nanjing University, 210093 Nanjing, China

## Abstract

The two-dimensional topological insulators host a full gap in the bulk band, induced by spin–orbit coupling (SOC) effect, together with the topologically protected gapless edge states. However, it is usually challenging to suppress the bulk conductance and thus to realize the quantum spin Hall (QSH) effect. In this study, we find a mechanism to effectively suppress the bulk conductance. By using the quasiparticle interference technique with scanning tunneling spectroscopy, we demonstrate that the QSH candidate single-layer 1*T*’-WTe_2_ has a semimetal bulk band structure with no full SOC-induced gap. Surprisingly, in this two-dimensional system, we find the electron–electron interactions open a Coulomb gap which is always pinned at the Fermi energy (*E*_F_). The opening of the Coulomb gap can efficiently diminish the bulk state at the *E*_F_ and supports the observation of the quantized conduction of topological edge states.

## Abstract (web summary)

The conductance from bulk bands in a topological insulator usually blurs effects arising from edge states. Here, Song et al. report a Coulomb gap opened by electron–electron interactions, which effectively suppress the bulk conductance and promote observation of topological edge states in the single-layer 1*T*’-WTe_2_.

## Introduction

The two-dimensional topological insulators (2DTIs) show great potentials in future applications, such as low dissipation electronics and quantum computing^1–4^. Since the discovery of quantum spin Hall (QSH) effect in HgTe/CdTe quantum wells^5,6^, enormous efforts have been devoted to practically useful 2DTI materials^5–21^. Qian et al.^11^ predicted a class of QSH materials in the single-layer 1*T*’-phase of transition-metal dichalcogenide (TMD), TX_2_, where T represents a transition-metal atom (Mo, W) and X stands for a chalcogen atom (S, Se, or Te). The band inversion happens between transition-metal *d* orbitals and chalcogenide *p* orbitals, and the SOC interaction further opens a fundamental band gap^11^. Recently, great experimental progress have been made in the single-layer 1*T*’-WTe_2_^22–26^. For example, the transport measurement on the exfoliated monolayer WTe_2_ sheet showed the existence of topological edge conductance^22^. The natural single-layer 1*T*’-WTe_2_ was successfully grown by molecular beam epitaxy (MBE) technique^23,24^, and its topological edge states were also visualized by scanning tunneling microscopy/spectroscopy (STM/STS) measurement^23,24^. Quantized edge conductance has been realized in the single-layer WTe_2_ at temperatures up to 100 K^25^.

However, the experiments showed that the single-layer 1*T*’-WTe_2_ exhibits an insulating behavior at low temperature^22–24^, inconsistent with the semimetal bulk band structure as initially predicted by DFT under single-electron frame^11^. Even though several possible mechanisms have been suggested to explain this insulating behavior^23,24,27,28^, the issue still remains controversial because of the lack of fully understanding of its electronic structure. The STS-QPI technique is suitable and crucial to solve such a controversy, since it has the capability to characterize the band structure with high energy-resolution, for both the occupied and unoccupied states near the Fermi energy *E*_F_.

In this study, we employ the QPI-STS/STM to detailedly investigate the electronic structure of single-layer 1*T*’-WTe_2_. At first, we clarify that the conduction bands (Te *p*) cross the Fermi level along the Y–Γ–Y direction, and the energy of their bottoms is lower than the top of the valence band (W *d*), in agreement with the DFT calculation under the single-electron frame^11^. Second, we explicitly reveal that there is a Coulomb gap at the Fermi level, which arises from the electron interactions in the 2D system rather than the spin–orbit coupling (SOC). Unlike the SOC-induced gap, as generally considered in 2DTIs, the Coulomb gap discovered in this study always locates at the Fermi level, independent of the electron doping. This exotic gap in the single-layer 1*T*’-WTe_2_ can efficiently filter its topological edge channels directly from the vanishing bulk states at the Fermi level, regardless of the gap size.

## Results

### Topography and spectroscopy measurement

**Fig. 1:**
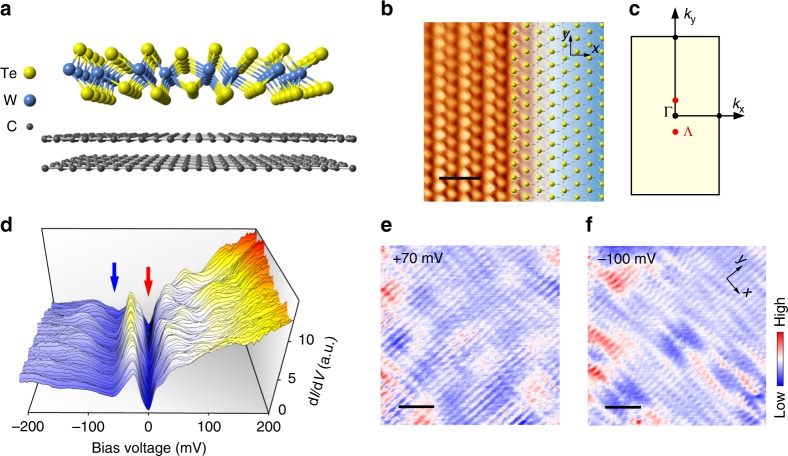
STM topography and d*I*/d*V* spectra of single-layer 1*T*’-WTe_2_ grown on BLG/SiC(0001). **a** Atomic model of the single-layer 1*T*’-WTe_2_ structure grown on bilayer graphene /SiC substrate. **b** Atomic resolution STM image of the single-layer 1*T*’-WTe_2_ surface (5 × 5 nm^2^, *U* = + 15 mV, *I*_t_ = 300 pA). The length of the scale bar is 1.0 nm. The right half of the STM image is overlaid by the lattice of 1*T*’-WTe_2_ with the top-layer Te atoms represented by yellow balls. **c** The corresponding Brillion Zone of single-layer 1*T*’-WTe_2_. The locations of the minimum of conduction band are marked by red dots and labeled by Λ. **d** Spatial variation of d*I*/d*V* spectra (*U* = + 100 mV, *I*_t_ = 200 pA) taken at 128 locations along a line of ~16 nm. The red and blue arrows mark the energy gap at *E*_F_, and the intensity minimum. **e**, **f** Two typical d*I*/d*V* maps (18 × 18 nm^2^) taken on the single-layer 1*T*’-WTe_2_ terrace. *U* = + 70 mV for **e** and *U* = −100 mV for **f**, *I*_t_ = 100 pA. The length of the scale bar is 3.6 nm. The QPI-induced spatial modulations are clearly identified

The single-layer 1*T*’-WTe_2_ possesses a sandwich structure with three atomic layers of Te–W–Te^29,30^, as shown in Fig. 1a. The 1*T*’-WTe_2_ phase is formed due to the spontaneous lattice distortion in the 1*T* structure where the W–Te–W stacks in the rhombohedral (ABC) atomic-layer order. The distorted W atoms in the *x* axis form the one-dimensional (1D) zig-zag atomic chains along the *y* axis and a doubling 2 × 1 periodicity^31^. It should be noted that the topmost Te atoms are not in a plane due to the distortion of the W atoms underneath. The atomic resolution STM image of the single-layer 1*T*’-WTe_2_, as shown in Fig. 1b, discloses the exact atomic registry and the apparent atomic height, which is consistent with the 1*T*’ phase atomic structure. The corresponding reciprocal Brillouin Zone is shown in Fig. 1c.

The local density of states (LDOS) in a wide bias range (*U* = ±1.0 V), as represented by the differential conductance d*I*/d*V* spectrum in Supplementary Figure 1, agrees well with the previous reports^23,24^ and the DFT calculated band structure for the freestanding monolayer^11^. A series of differential conductance d*I*/d*V* spectra (128 curves in total) in a smaller bias range, taken along a line of ~16 nm, are plotted together in Fig. 1d. The key features in these d*I*/d*V* curves are the energy gap (red arrow) at the *E*_F_ and the kink at approximately −60 mV (blue arrow). These two features are uniform and unchanged in real space. As will be discussed later, the energy gap is always located at the *E*_F_ and caused by the Coulomb interaction between electrons, and the kink is related to the intensity minimum of the overlap region between valence and conduction bands rather than a full band gap. The features that vary along the surface come from the QPI, see particularly the bumps in the positive bias region in Fig. 1d. The d*I*/d*V* maps are measured over the 1*T*’-WTe_2_ terrace, and two typical maps are depicted in Fig. 1e, f, from which the spatial modulations due to the QPI of electronic Bloch waves can be clearly identified.

### Quasiparticle interference analysis

**Fig. 2:**
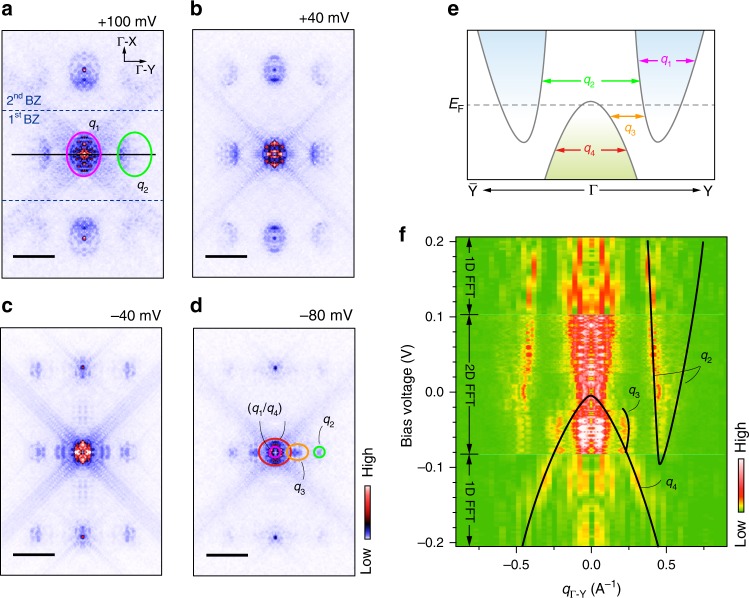
QPI patterns and the FFT images at different bias energies. **a**–**d** Series of FFT images transformed from the measured d*I*/d*V* maps, with the bias voltages of +100, +40, −40, and −80 mV respectively. The length of the scale bar is 0.5 Å^−1^. The d*I*/d*V* maps are measured on the same region as shown in Fig. 1e, f. The colored ovals represent the scattering *q* vectors as indicated in **e**. All the FFT images are symmetrized and drift-corrected. **e** Schematic band structure along the Y–Γ–Y direction. The intra- and inter-band scatterings are marked by the red arrows. The *q*_1_ represents the intra-band scattering of the conduction band, which forms the pink ovals (**a**–**d**). The *q*_2_ represents the inter-band scattering between the two conduction bands, which forms the green ovals in **a**–**d**. The *q*_3_ represents the inter-band scattering between the valence band and conduction band, which forms the orange oval in **c** and **d**. The *q*_4_ represents the intra-band scattering of the valence band, which forms the red oval in **d**. **f** E-q dispersion along the Y–Γ–Y direction. In the region labeled by 2D-FFT, from −80 to +100 mV, the dispersion is extracted from the line-cut profiles in the 2D-FFT images in the Supplementary Figure 5. The position of the line-cut is marked by the black line in **a**. In the regions labeled by 1D-FFT, from −200 to −80 mV and from +100 to +200 mV, the dispersion is extracted from the 1D-FFT of the d*I*/d*V* spectroscopic map taken along the *y* axis (See Supplementary Figure 6). The black lines schematically illustrate the band dispersion of *q*_2_, *q*_3_, and *q*_4_

Figure 2a–d shows the fast Fourier transform (FFT) of the d*I*/d*V* maps taken at different bias voltages, sweeping from the unoccupied to the occupied states. More QPI patterns can be found in the Supplementary Figures 2–5. The wave vector *q* = *k*_f_ − *k*_i_ obtained from the QPI pattern can be understood as the elastic scattering of electronic Bloch waves, from the initial state of *k*_i_ to the final state of *k*_f_. In the First Brillion Zone of Fig. 2a, b (the middle part), there exist three ellipses in all the QPI patterns, with one located at the center and the other two symmetrically located along the Y–Γ–Y direction, as guided by the pink and green ovals. In Fig. 2c, d, two extra features start to appear and are symmetrically located along the Y–Γ–Y as well, as marked by the orange oval. The replicas of these features with weaker intensity are found in the second Brillion Zone, i.e. the upper and lower parts in Fig. 2a–d. Figure 2e shows the schematic band structure derived from the previous DFT calculation (see Fig. S1F of ref. ^11^). Different from its bulk counterpart of *T*_d_-WTe_2_, the single-layer 1*T*’-WTe_2_ still holds the inversion symmetry^32^. In the presence of weakly coupling between the epitaxial WTe_2_ and BLG/SiC, the inversion symmetry may be broken. However the substrate effect is negligible in this work, as indicated by the previous ARPES measurement that showed no prominent band splitting ^23^. Therefore the bands are still spin-degenerate. Comparing Fig. 2a–d with Fig. 2e, one can find that the central pocket comes from two scattering channels: the intra-band scattering of the conduction band (pink oval in Fig. 2a, *q*_1_ in Fig. 2e) and that of the valence band (red oval in Fig. 2d, *q*_4_ in Fig. 2e). These two channels are distinguishable when the energy is far away from the *E*_F_, and entangled with each other when close to each other. The size of the two side ellipse pockets (green ovals) shrinks with decreasing bias voltage, confirming that these ellipses are associated with the electron-like pocket scattering. According to Fig. 2e, they can be assigned as the scattering channel of *q*_2_, i.e., the inter-conduction band scattering. The extra features as marked by the orange oval can be assigned as the inter-band scattering between the valence and conduction bands (*q*_3_ in Fig. 2e).

The E-q dispersion, namely the scattering band structure, can be extracted from the line cuts taken on the QPI patterns along some specific directions. In Fig. 2f is plotted the E-q dispersion along the Y–Γ–Y direction. The QPI data that are used to extract the E-q dispersion are shown in the Supplementary Figures 5 and 6. For guiding eyes, the black lines are drawn in Fig. 2f to track the dispersions of *q*_2_, *q*_3_, and *q*_4_, respectively. One can see that there exists an energy overlap region between the valence band and the conduction bands, which suggests a semimetal band structure. It is worthwhile noting that our STS results look similar to the previous study^23^, in particular the kink feature below Fermi energy. However, our high-resolution QPI analysis indicates that this kink feature is not caused by the opening of a full SOC gap, but corresponds to the intensity minimum of DOS in the band overlap region.

**Fig. 3:**
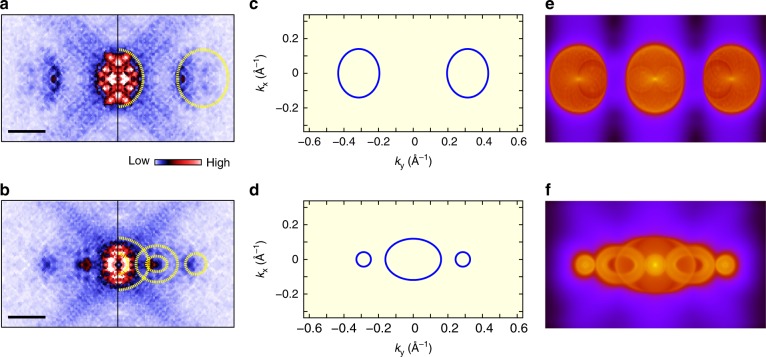
Comparison of the QPI patterns with the DFT simulation. **a**, **b** The fast Fourier transform (FFT) images transformed from the d*I*/d*V* maps taken at +70 and −80 mV, respectively. The length of the scale bar is 0.3 Å^−1^. The FFT images are symmetrized and drift-corrected. Only the patterns in the first Brillion Zone are shown. The overlaid yellow dashed ovals briefly outline the QPI patterns. **c**, **d** Schematic constant energy contours at *E* = +100 mV and *E* = −70 mV, respectively. **e**, **f** The DFT-simulated QPI patterns based on **c** and **d**

To further understand the physical origins for the QPI wave vectors, we compare the experimental results with our DFT-simulated patterns. Two typical zoom-in QPI patterns are shown in Fig. 3a, b, with the former energy cutting only the conduction band and the latter one cutting the band overlap region. The corresponding schematic constant energy contours (CECs) are plotted in Fig. 3c, d, from which the simulated QPI patterns are obtained in terms of the DFT calculation, as shown in Fig. 3e, f. In both cases, the experimental QPI results are consistent with the simulated QPI patterns. Such a good agreement further confirms the validity of the band structure model of Fig. 2e and the origins of these QPI features. In particular, the existence of *q*_3_ corresponding to the scattering between the valence and conduction bands provides a decisive evidence for the semi-metallic band structure without a full band gap. This semimetal band structure of single-layer 1*T*’-WTe_2_ is also in good agreement with the DFT calculation under single-electron frame^11^. As a result, such experimental results unambiguously show that the insulating behavior in single-layer 1*T*’-WTe_2_ is not caused by a single-particle band gap.

### Potassium doping

**Fig. 4:**
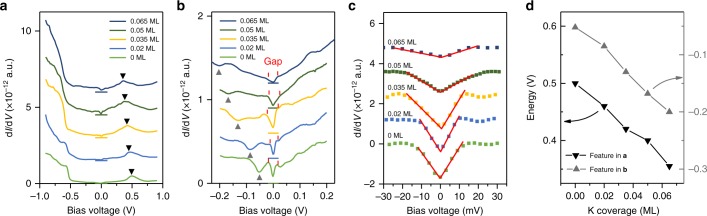
d*I*/d*V* spectra taken on the 1*T*’-WTe_2_ surface with different potassium (K) coverage. **a**, **b** d*I*/d*V* spectra taken in a large and small energy scales. *U* = +500 mV for **a** and *U* = +200 mV for **b**, *I*_t_ = 200 pA. The black colored triangles in **a** and gray colored triangles in **b** mark the characteristic features in the d*I*/d*V* spectra, which move towards the left upon K deposition and are used to determine the shift of Fermi energy. The gap region is marked by the red vertical lines in **b**. **c** The Zoom-in spectra of **b** showing the DOS near the Fermi energy. The suppression of the DOS at the Fermi level is always distinguishable. The background is subtracted, and the DOS is fitted with a linear function near the Fermi energy. **d** The position of the characteristic features as marked in **a** and **b** as a function of K coverage

We next turn to investigate the puzzling gap at the Fermi level. Our tunneling spectroscopy study confirms that there is a soft gap at the Fermi level. However, the above QPI analysis indicates that the band structure of single-layer 1*T*’-WTe_2_ is semimetal, without a full gap between the conduction and valance bands. We thus believe that such a gap at the Fermi level is not attributed to the SOC-induced single-particle gap. The possibility of the superconducting gap, should be excluded, because the previous transport study indicated an insulating behavior at low temperature^24^. Furthermore, the gap cannot be suppressed by applying magnetic field (Supplementary Figure 7). To clarify the mechanism for the gap formation, we purposely dope electrons to the single-layer 1*T*’-WTe_2_ surface via potassium (K) deposition, to tune the position of Fermi level. Figure 4a and b shows series of d*I*/d*V* spectra of the single-layer 1*T*’-WTe_2_ with different coverage of surface K. The corresponding surface morphologies can be found in the Supplementary Figure 8. The features as marked by triangles in Fig. 4a, b can be used to determine the energy shift of the Fermi level, and their dependence on the K coverage is plotted in Fig. 4d. The maximal upward shift of the Fermi level is ~150 mV. Surprisingly, as shown in Fig. 4b, c, the energy gap is always pinned at the *E*_F_ regardless of the position of *E*_F_, indicating that the gap may arise from the electron–electron Coulomb interaction. Such a gap can persist at elevated temperatures up to ~75 K, as demonstrated in our tunneling spectroscopy measurement of Supplementary Figure 9.

## Discussion

As early as the 1970s, it was proposed that in localized systems the long-range Coulomb interactions between electrons diminish the single-particle DOS in the vicinity of the Fermi level, and thus a soft gap in the DOS is formed, which was called the Coulomb gap^33–37^. In the 2D case, the DOS near the Fermi energy can be qualitatively given as:^37,38^

1 $$G\left( \varepsilon \right) \propto \left( {2/\pi e^4} \right)\left| \varepsilon \right|,$$

at *T* = 0 K, where *ε* represents the energy with respect to the Fermi energy *E*_F_. The Coulomb gap in the DOS can be observed experimentally at low enough temperatures, such that thermal excitations do not wash it out. Due to its sufficient decoupling from the BLG/SiC substrate^24^, the single-layer WTe_2_ is expected to form an ideal 2D localized electron system. The distorted W atomic zig-zag chains further enhance the anisotropy of localization. For our tunneling spectroscopy measurement, the d*I*/d*V* curves near the *E*_F_ can be fitted very well with the linear format of a soft gap, as shown in Fig. 4c^37,38^. Considering the case that the gap is in a linear shape and always pinned at the *E*_F_, a Coulomb gap is strongly suggested.

Basically, the Coulomb gap can be understood as a result of exitonic attraction of electrons and holes near the Fermi level, which depletes the one-particle DOS there^38^. Consider a transfer of one electron from a site *i* that is occupied in the ground state to a site *j* that is vacant in the ground state. The resulting energy change is given by Δ*E* = *E*_*j*_ − *E*_*i*_ – *e*^2^/*r*_*ij*_, where *E*_*j*_ (*E*_*i*_) is the single-particle energy at site *i* (*j*) and the last term describes the exitonic effect, i.e., the Coulomb interaction of the created electron–hole pair^32^ with *r*_*ij*_ the distance between the sites *i* and *j*. According to the stability criteria of the ground state, Δ*E* must be positive. It can be shown^35^ that the concentration of such sites, n(*ε*), cannot exceed (*ε*/e^2^)^2^ in the 2D case. Thus, the 2D single-particle DOS, G(*ε*) = dn(*ε*)/d*ε*, is proportional to *ε*, vanishing when ε tends to zero at least as fast as ε.

Localized and interacting electrons / holes are necessary to form a Coulomb gap. According to the theory of two-dimensional Anderson localization, all quantum states of an infinitely large disordered sample are localized even for a vanishingly small but finite disorder. Such a small disorder is always present in our samples, and so the localization is not surprising. Indeed, the semiconductor-like conductivity behavior observed in the previous transport measurement^24^ did confirm the localization.

The single-layer WTe_2_ is weakly coupled to the substrate and forms a 2D system. As determined by the QPI characterization, the Fermi level of the undoped single-layer 1*T*’-WTe_2_ crosses the two conduction bands along the Y–Γ–Y direction, and cuts the valence band in proximity of its top. Similar to the bulk WTe_2_, the electron pocket in the conduction band and the hole pocket in the valence band make the single-layer WTe_2_ a semimetal. The balanced electron–hole compensation means that each electron has a corresponding hole. It can be thus expected that the excitonic effect in single-layer WTe_2_ is prominent. Even though other possible mechanisms cannot be excluded, all of our experimental results agree well with the Coulomb gap origin. In all the cases, the Coulomb gap appears at *E*_F_, in the conduction bands and/or in the valence band. The opening of Coulomb gap results in the insulating behavior of 1*T*’-WTe_2_ at low temperature.

Comparing to the SOC-induced negative gap of ~0.11–0.13 eV^11^, the Coulomb gap observed is much smaller and not likely to change the topology of the system. Furthermore, the topological edge states are not expected to be influenced as well, due to its dimensionality. The topologically nontrivial edge states are closely related to the SOC. The band inversion and large SOC effect is key to the quantum spin Hall state in monolayer 1*T*’-WTe_2_, as predicted by Qian et al.^11^ The Coulomb gap is essentially different from the SOC gap. It is not a real band gap, but only attenuates the intensity of the density of state (DOS) near the Fermi energy. This Coulomb gap does not change the SOC effect, and the band inversion is still reserved. Therefore the system is still topologically nontrivial.

In summary, we have characterized the single-layer 1*T*’-WTe_2_ with high-resolution QPI-STS/STM, and verified a semimetal band structure, where there exists a band inversion near the Γ point, without a full SOC-induced bulk band gap. An important finding is the Coulomb gap induced by the electron interactions in this 2D localized electron system. The observation of the Coulomb gap in the single-layer QSH systems is vital to distinguish the topological edge states from the vanishing bulk band state and greatly facilitates the realization of QSH effect. Further in-depth experimental studies are strongly demanded.

## Methods

### Sample preparation and STM/STS characterization

The nearly freestanding single-layer 1*T*’-WTe_2_ film was prepared by MBE technique on the bilayer graphene (BLG) formed on the 6*H*-SiC(0001) substrate. The detailed procedure of the sample growth can be found elsewhere^24^. After the MBE growth, the sample was transferred immediately into the LT-STM (Unisoku Co., USM1600) for scan at ~4.2 K. Differential conductance d*I*/d*V* spectra were acquired through a standard lock-in technique with the ac modulation of ~3–5 mV at 996 Hz. Experimental QPI maps were generated by symmetrizing the Fourier transformed d*I*/d*V* maps.

## Electronic supplementary material

Supplementary Information

## Data Availability

The data that support the findings of this study are available from the article and Supplementary Information files, or from the corresponding author upon reasonable request.
