# Supplementary material for: Observation of Coulomb gap in the quantum spin Hall candidate single-layer 1T’-WTe2
Source: Nat Commun. 2018 Oct 4;9:4071. doi: 10.1038/s41467-018-06635-x (PMC6172222; doi:10.1038/s41467-018-06635-x)
Supplement: Supplementary file 1 — Supplementary Information [file 41467_2018_6635_MOESM1_ESM.pdf]

Observation of Coulomb gap in the quantum spin Hall candidate single-layer 1*T'*-WTe_2

Ye-Heng Song[1†], Zhen-Yu Jia[1†], Dongqin Zhang[1], Xin-Yang Zhu[1], Zhi-Qiang Shi[1], Huaiqiang Wang[1], Li Zhu[1], Qian-Qian Yuan[1], Haijun Zhang[1,2], Ding-Yu Xing[1,2], Shao-Chun Li[1,2*]

1. National Laboratory of Solid State Microstructures, School of Physics, Nanjing University, Nanjing 210093, China

2. Collaborative Innovation Center of Advanced Microstructures, Nanjing University, Nanjing 210093, China

[†] These authors contributed equally to this work.

* email: scli@nju.edu.cn

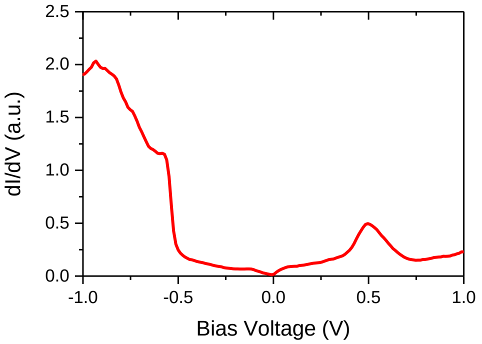

Supplementary Figure 1. d*I*/d*V* spectrum (*U*= +500 mV, I_t = 200 pA) taken on the single-layer 1*T*′-WTe_2 in the bias range from -1.0 V to +1.0 V.

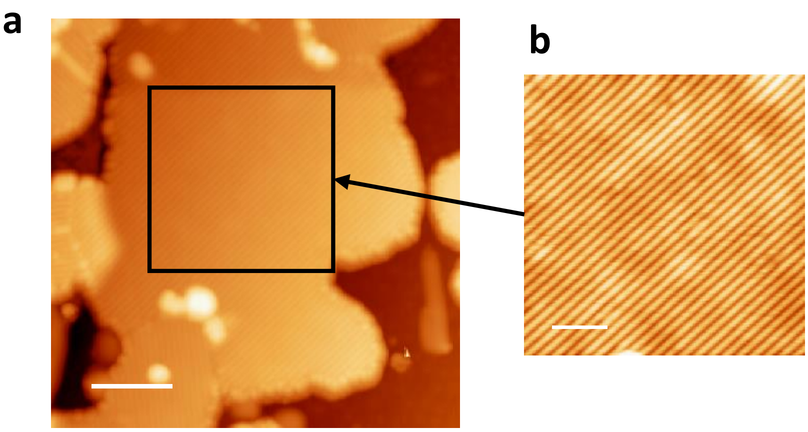

Supplementary Figure 2. STM topographic images of the monolayer $1T'$-WTe_2 where dI/ dV maps are taken. (a) STM image of the monolayer $1T'$-WTe_2 island (U = +1.0 V, I_t = 100 pA). The scale bar is 8 nm. The black square frame marks the area where the dI/dV maps are taken. (b) Zoom in area as marked in (a) (U = +0.2 V, I_t = 100 pA). The scale bar corresponds to 3.6 nm.

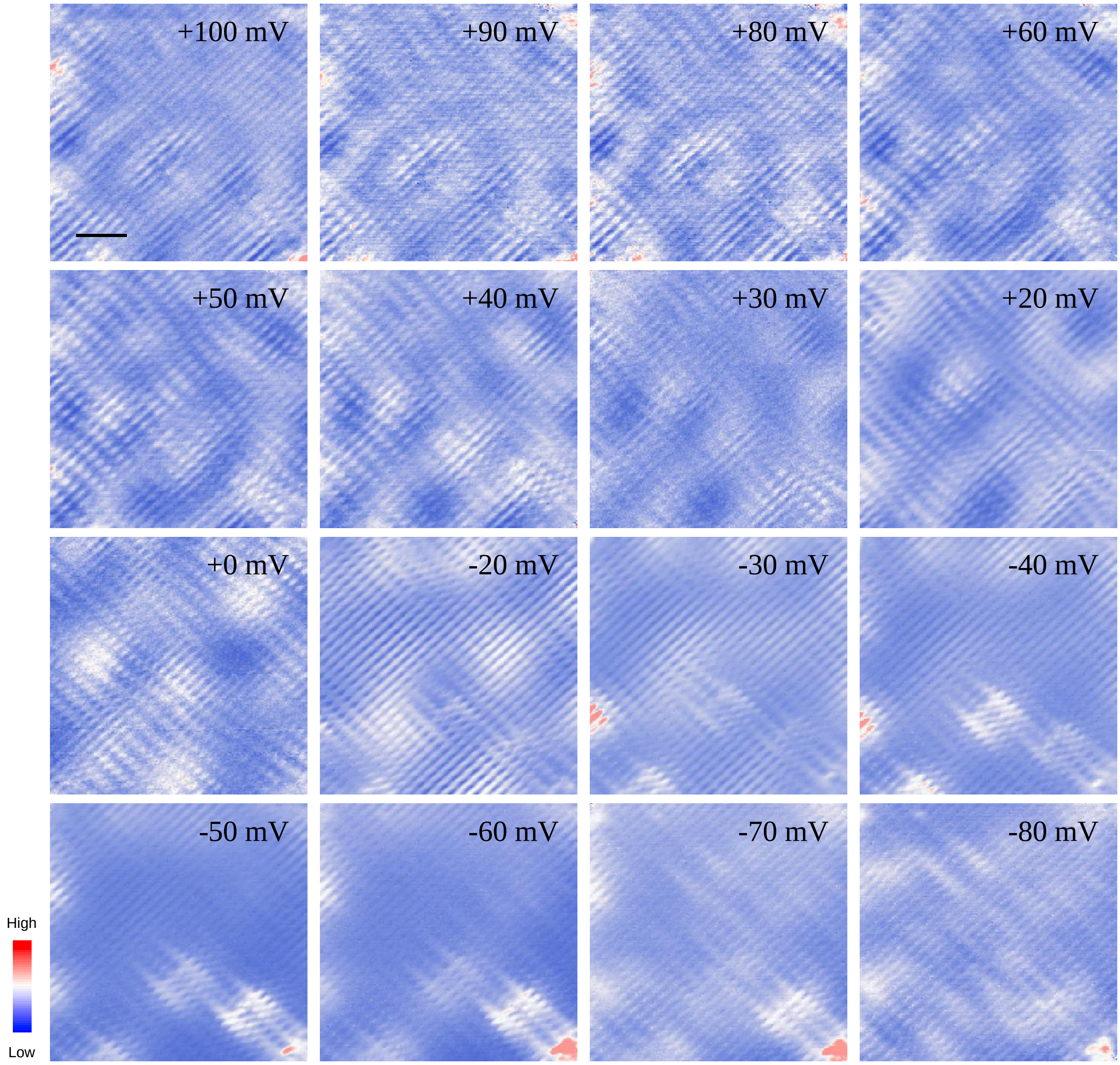

Supplementary Figure 3. The experimental dI/dV maps taken at various bias voltages on the region as marked in Supplementary Figure 2. The bias voltage is labeled in each image (each dI/dV map is taken at the bias as is labeled and I_t = 100 pA except for the map at 0 mV. The dI/dV map for 0 mV is taken at U = +30 mV and I_t = 100 pA. The scale bar corresponds to 3.6 nm.

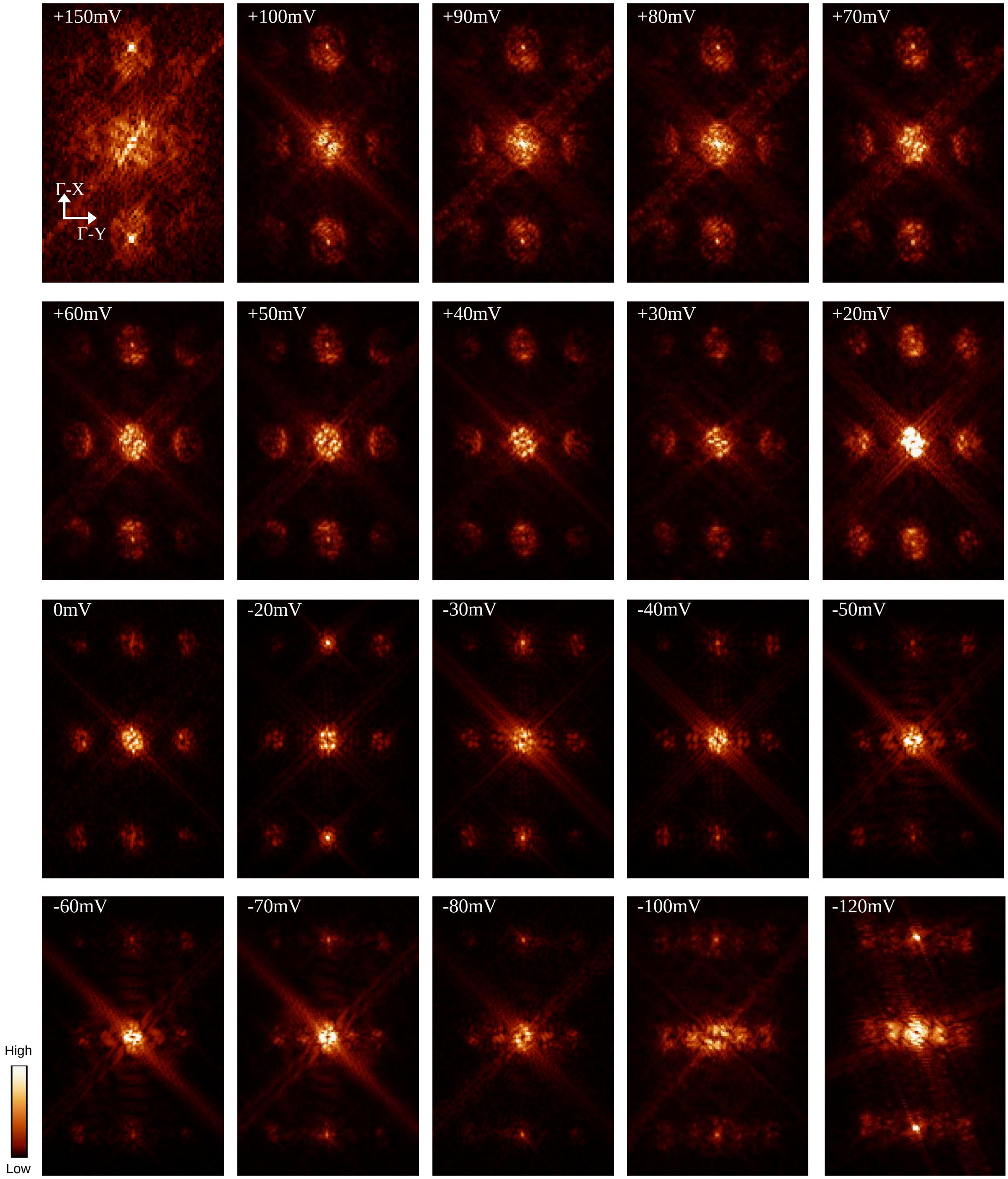

Supplementary Figure 4. FFT images (without symmetrization process) showing the evolution of the QPI patterns with bias voltage. The bias voltage is labeled in each image.

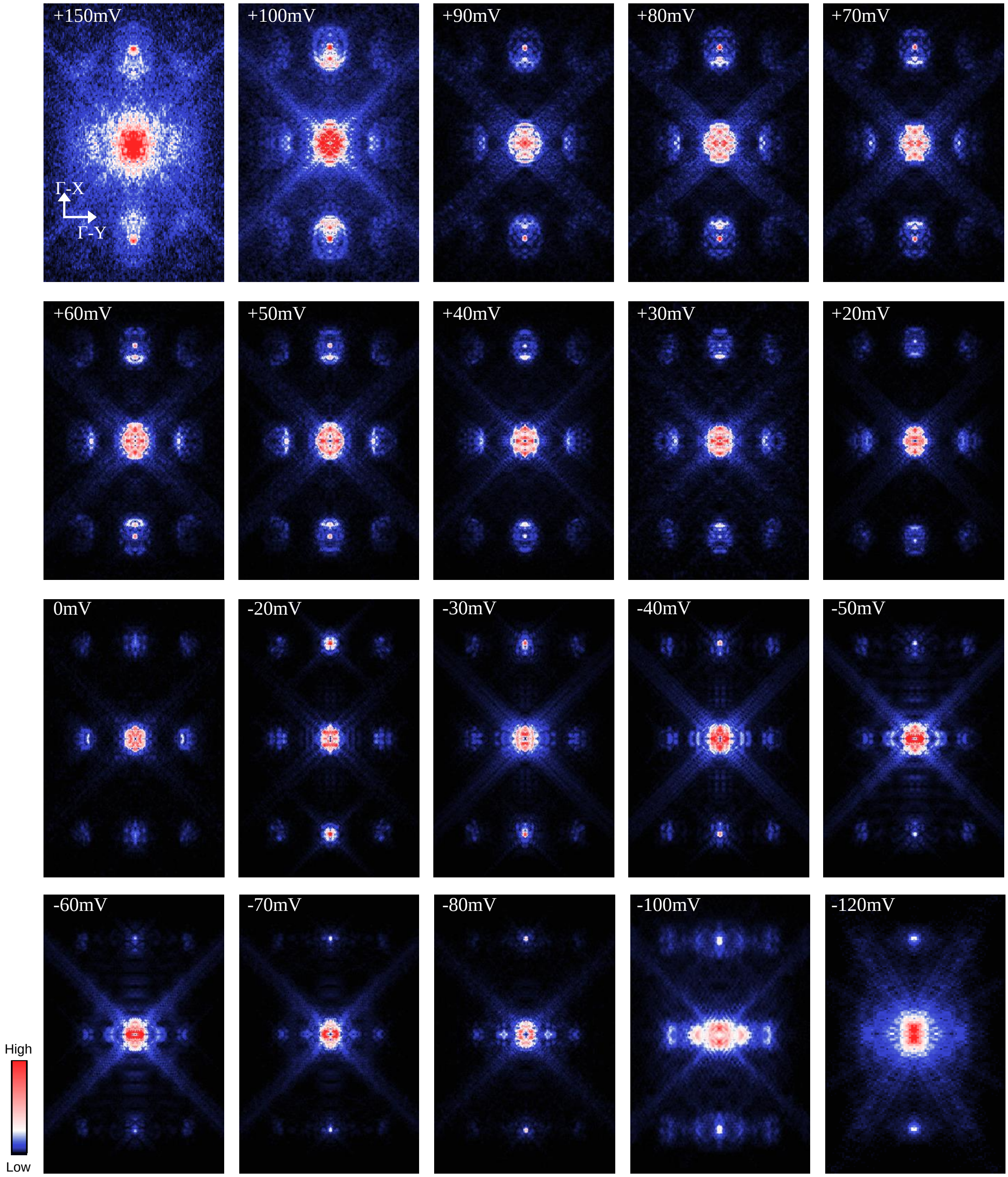

Supplementary Figure 5. FFT images showing the evolution of the QPI patterns with bias voltage. The bias voltage is labeled in each image. All the FFT images are symmetrized and drift-corrected.

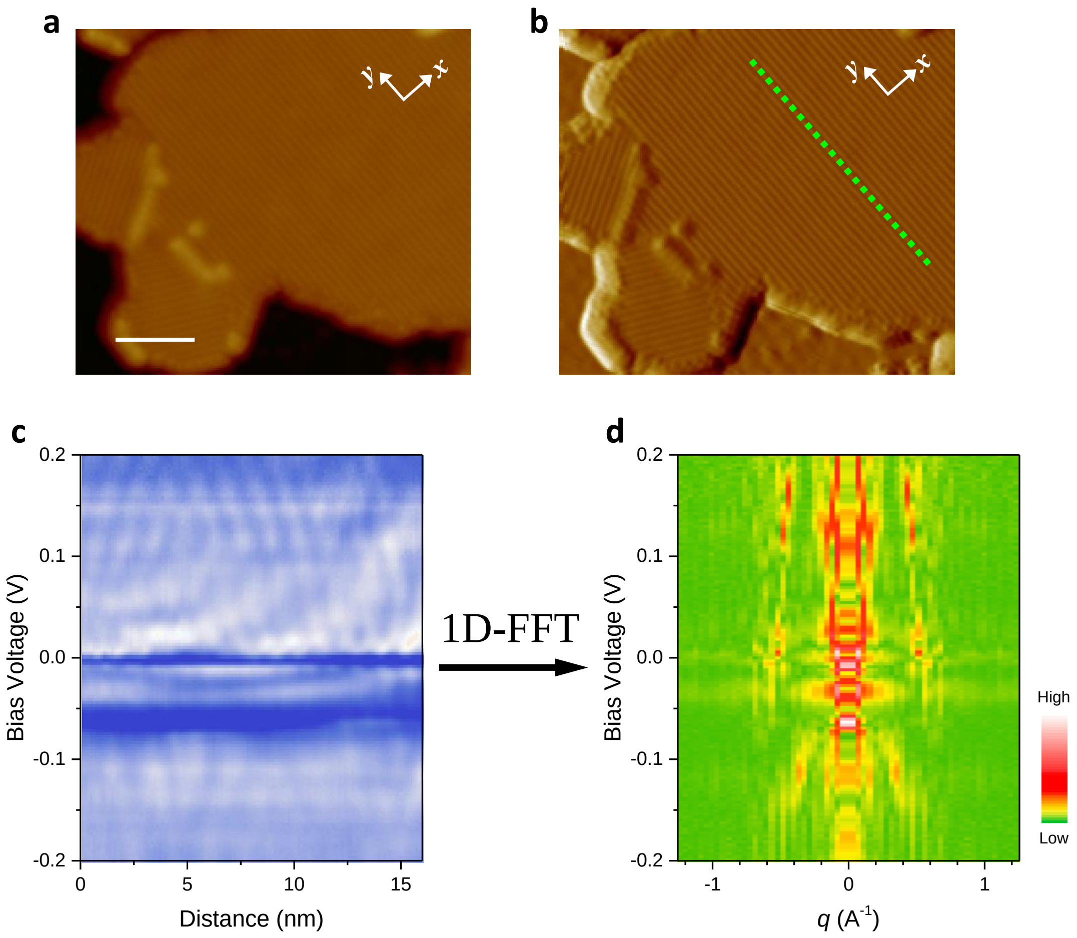

Supplementary Figure 6. Line-scan dI/dV spectra taken along the y-axis.
(a) STM topographic images (U = +1.0 V, I_t = 100 pA) of the area for the line-scan dI/dV spectroscopic measurement taken along the y-axis. The scale bar is 6.4 nm. (b) Differential images of (a) showing clearly the row direction. The green dotted line marks the positions where the line-scan dI/dV spectra are taken. (c) The corresponding dI/dV spectroscopic map (U = +100 mV, It = 200 pA) taken along the green line in (b). (d) The 1D FFT image of (c).

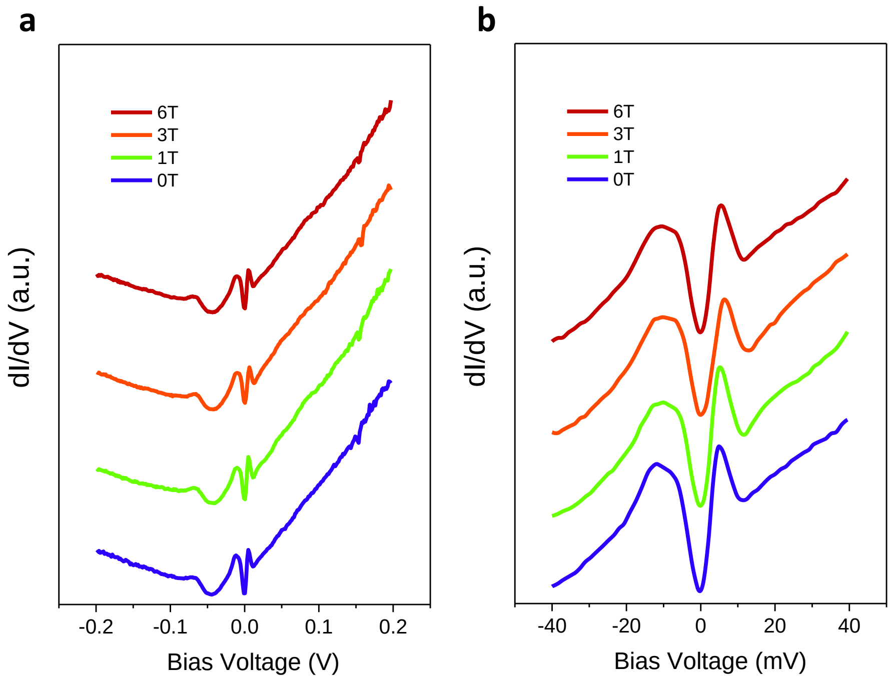

Supplementary Figure 7. Magnetic field dependence of dI/dV spectra. The dI/dV spectra are measured at a specific lattice site on the 1T'-WTe_2 terrace under the magnetic field up to 6 T. The magnetic field is applied perpendicular to the surface (along c axis). (a) Large energy scale dI/dV spectra (U = +100 mV, It = 300 pA) ; (b) Small energy scale dI/dV spectra (U = +40 mV, It = 200 pA).

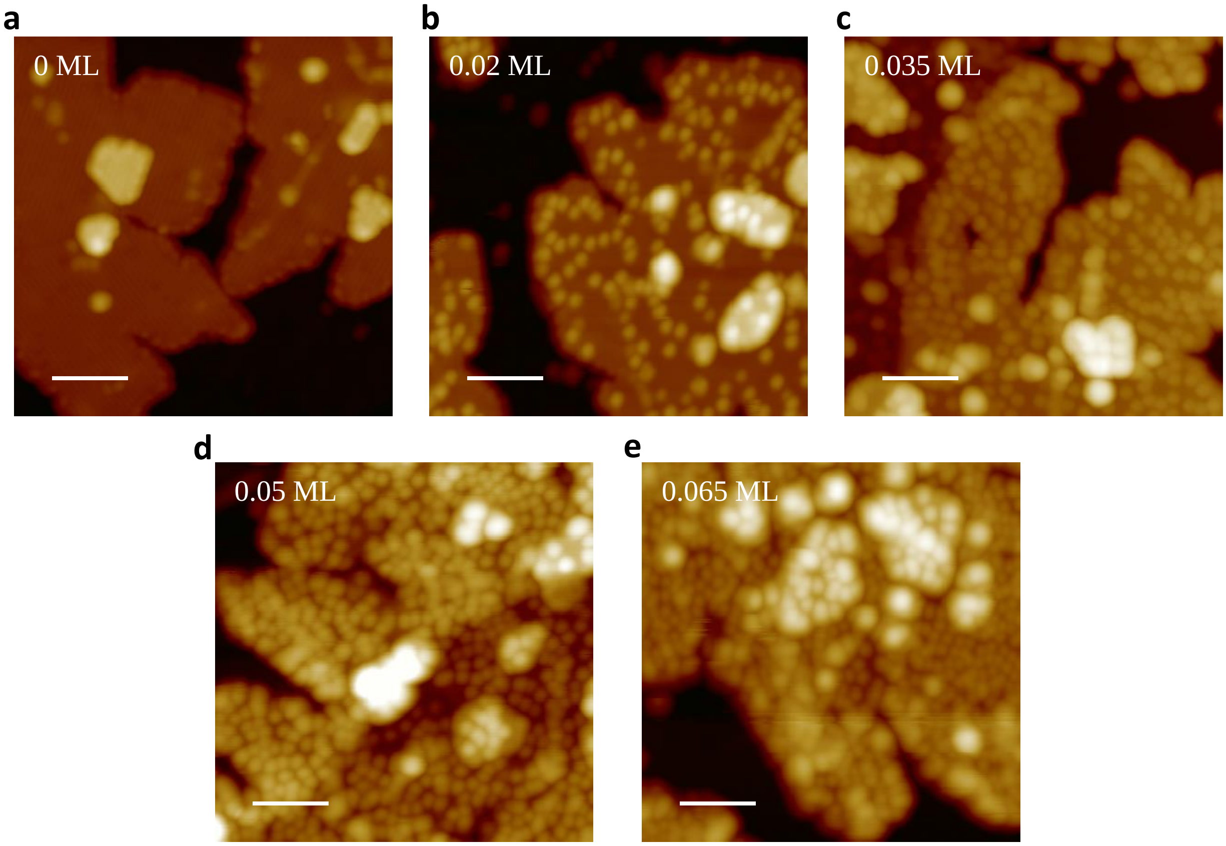

Supplementary Figure 8. Surface morphology of single layer 1*T*′-WTe_2 upon K deposition. (a) STM topographic image of the clean surface of 1*T*′ -WTe_2 islands (U = +2.0 V, I_t = 100 pA). The scale bar is 8 nm. (b)~(e) STM topographic images (U =+1.0 V, I_t = 100 pA) of 1*T*′-WTe_2 islands after potassium deposition. The scale bar is 8 nm. The coverage of the potassium is labeled in each image.

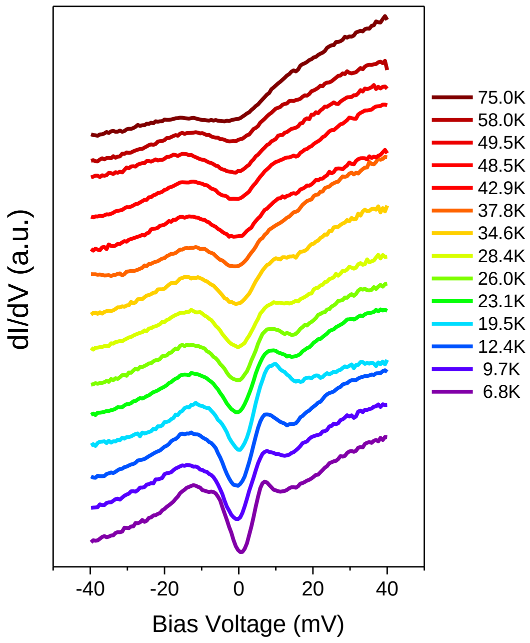

Supplementary Figure 9. Evidence of the existence of the gap at Fermi level at elevated temperatures up to ~75 K. The d*I*/d*V* tunneling spectra (U = +40 mV, I_t = 200 pA) are taken at a specific lattice site on the 1*T*′-WTe_2 terrace. The gap shape at the Fermi level can be clearly identified at temperatures up to ~75 K.

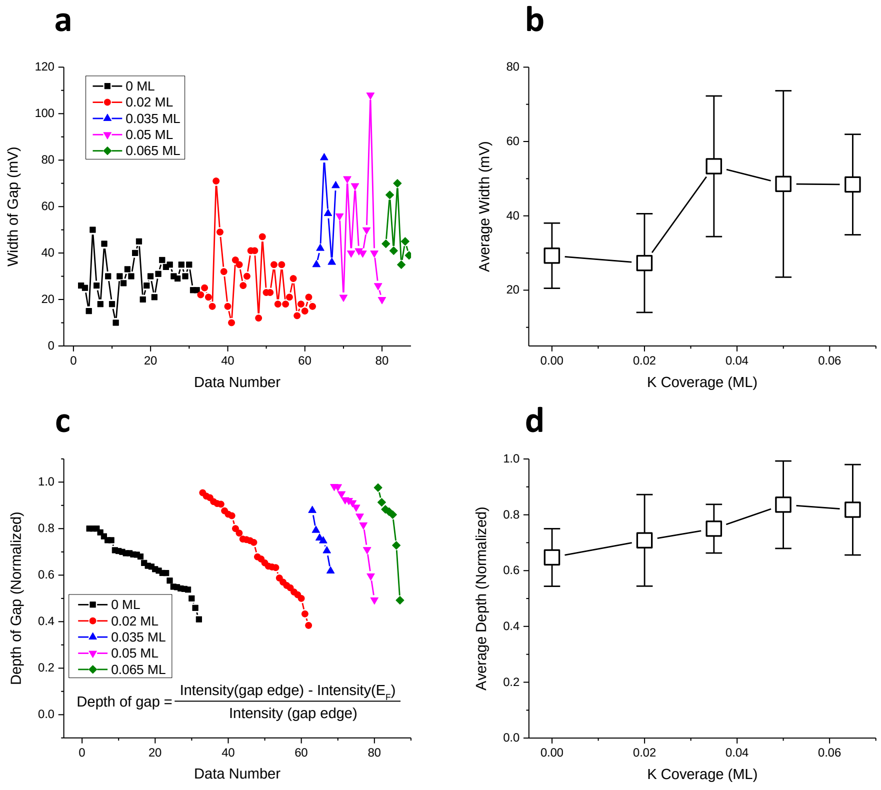

Supplementary Figure 10. Statistical results of the gap width and depth at various K coverage. The depth is defined as dI/dV (E_F) / dI/dV (gap edge). (a) Measured gap width at various K coverage. Each dot represents a measured value on a dI/dV spectrum. The different colors mark the different K coverage. (b) Average width of the gap at various K coverage. The average gap width shows a trend to increase. (c) Measured gap depth at various K coverage. Each dot represents a calculated value on a dI/dV spectrum. The different colors mark the different K coverage. (d) Average depth of gap at various K coverage. The average gap depth shows a trend to decrease, meaning the gap becomes deeper.

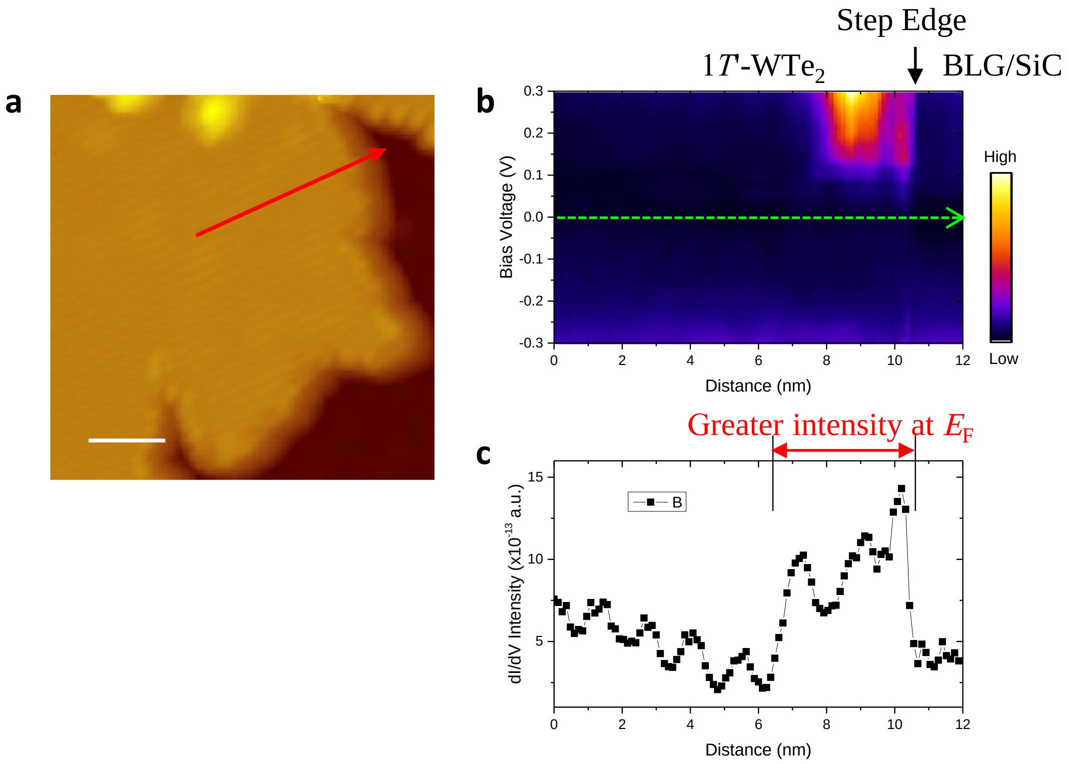

Supplementary Figure 11. Charaterization of the edge state at the 1T'-WTe_2 step edge. (a) STM image (U = +1.0 V, I_t = 100 pA) of the single-layer 1T'-WTe_2 island grown on Graphene/SiC substrate. The scale bar corresponds to 4 nm. (b) dI/dV spectra (U = +200 mV, I_t = 200 pA) taken along the red arrowed line in (a) and shown in 2D color mode. The x axis represent the location, and the y axis the Bias voltage. The dI/dV intensity is represented by the colored map. (c) The line-cut profile taken along the green arrowed line in (b) showing the location dependent dI/dV intensity at Fermi energy.
